# Inverse probability of treatment-weighted competing risks analysis: an application on long-term risk of urinary adverse events after prostate cancer treatments

**DOI:** 10.1186/s12874-017-0367-8

**Published:** 2017-07-10

**Authors:** Charlotte A. Bolch, Haitao Chu, Stephanie Jarosek, Stephen R. Cole, Sean Elliott, Beth Virnig

**Affiliations:** 1 University of Minnesota, Twin Cities Campus, 3 Morrill Hall, 100 Church St. S.E, Minneapolis, MN 55455 USA; 2 University of North Carolina, Chapel Hill, 2105E McGavran-Greenberg Hall, CB #7435, Chapel Hill, NC 27599 USA; 3 Present address: 5055 SW 9th Lane, Gainesville, FL 32607 USA

**Keywords:** Prostate cancer, Survival analysis, Competing risks, Prostate cancer, Inverse probability weighting, Confounding bias, Urinary adverse events

## Abstract

**Background:**

To illustrate the 10-year risks of urinary adverse events (UAEs) among men diagnosed with prostate cancer and treated with different types of therapy, accounting for the competing risk of death.

**Methods:**

Prostate cancer is the second most common malignancy among adult males in the United States. Few studies have reported the long-term post-treatment risk of UAEs and those that have, have not appropriately accounted for competing deaths. This paper conducts an inverse probability of treatment (IPT) weighted competing risks analysis to estimate the effects of different prostate cancer treatments on the risk of UAE, using a matched-cohort of prostate cancer/non-cancer control patients from the Surveillance, Epidemiology and End Results (SEER) Medicare database.

**Results:**

Study dataset included men age 66 years or older that are 83% white and had a median follow-up time of 4.14 years. Patients that underwent combination radical prostatectomy and external beam radiotherapy experienced the highest risk of UAE (IPT-weighted competing risks: HR 3.65 with 95% CI (3.28, 4.07); 10-yr. cumulative incidence = 36.5%).

**Conclusions:**

Findings suggest that IPT-weighted competing risks analysis provides an accurate estimator of the cumulative incidence of UAE taking into account the competing deaths as well as measured confounding bias.

**Electronic supplementary material:**

The online version of this article (doi:10.1186/s12874-017-0367-8) contains supplementary material, which is available to authorized users.

## Background

Prostate cancer is the second leading cause of death in men living in the United States with about 220,800 new cases diagnosed in 2015 as estimated by the American Cancer Society [1]. Based on 2011 data from SEER, the prevalence of prostate cancer in the United States was estimated to be 2,707,821 cases. Cancer diagnosis localized to the prostate gland usually indicates a better chance of survival compared to finding of regional or distant cancer spread. Prostate-specific antigen (PSA) screening has led to earlier detection of prostate cancer such that 81% of patients are diagnosed at the localized stage and the 5-year survival approaches 100% [2].

Significant medical and clinical emphasis is placed on prostate cancer diagnosis and treatment such as radiation therapy or surgery [3–5]. However, limited information is available to patients and physicians regarding subsequent urinary adverse events (UAEs) for different treatment options and their combinations. Examples of UAEs include urethral blockage due to scar tissue and bladder bleeding due to radiation damage. Knowing the risk of such events in advance can help patients and providers select the right treatment. In Jarosek et al., the authors conducted a matched-cohort design to compare differences in long-term severe UAEs post-treatment for prostate cancer patients compared to non-cancer controls using inverse probability of treatment (IPT) weighted Cox proportional hazard models to estimate the hazard ratio of severe UAEs [6]. Prostate cancer treatment options included: external beam radiotherapy, brachytherapy, radical prostatectomy, cryotherapy, and the combinations brachytherapy + external beam radiotherapy and radical prostatectomy + external beam radiotherapy.

Numerous articles have compared various competing risks analysis versus standard Cox proportional hazard regression, the correct calculation of cumulative incidence functions, and example applications to clinical research studies [7–9]. Bekaert et al. developed a marginal structural subdistribution hazard model to accommodate high dimensional and time-varying confounders and modeled the cumulative incidence function of ICU death [10]. The estimation of the standardized risk difference and ratio using inverse probability weights with the cumulative incidence function was explained in Cole et al. focusing on the effect of injection drug use on the risk of acquired immunodeficiency syndrome (AIDS) after initiation of antiretroviral therapy [11]. In observational research, confounding bias is a central concern and inverse probability (IP) weighting is commonly used to control for such bias due to measured variables [12, 13]. However, to the best of our knowledge, no one has considered IP-weighted competing risks analysis to account for confounding bias and competing events simultaneously for the estimation of the cumulative incidence of UAE [14, 15]. One obstacle is the lack of appreciation for the benefit of competing risks analysis in combination with IP weighting.

The use of competing risks analysis is used within the field of medicine due to its ability to correctly estimate the probability of failure from multiple competing causes rather than just one cause of interest [15–19]. In a competing risks analysis, the data used is the observed time to failure and an indicator of the type of competing risk events that occurred for each individual subject [20]. In Jarosek et al., the main event of interest was the incidence of the first post-treatment UAE, while death was considered as a censored event. Jarosek and colleagues adjusted confounding bias using IP weighting, but did not consider IP-weighted competing risks analysis. The use of IP-weighted competing risks analysis will enable the estimation of the cumulative incidence of UAEs adjusting for confounding bias and competing death, and thus providing a more accurate estimate of UAE disease burden [7, 8, 21, 22]. Due to recent developments in statistical software, one can easily implement IP-weighted competing risks analysis using PROC PHREG in SAS version 9.4 TS1M2 (SAS Institute, Inc., Cary, NC, USA).

In this article, we re-analyze the prostate cancer SEER-Medicare dataset in Jarosek et al. using IPT-weighted competing risks analysis taking into account the competing event of death and adjusting for confounding bias. We will also use a hypothetical example (see Additional file 1: Appendix A) to illustrate the detailed calculations of the various methods and highlight the differences between the multiple methods for estimating the cumulative incidence function [23].

### Study sample

The dataset of a matched cohort of elderly men diagnosed with non-metastatic prostate cancer and men without cancer was defined in the article by Jarosek et al. Specifically, the cases were selected from the SEER cancer registry data that can be linked to the Medicare claims database to provide information regarding long-term follow-up data for UAEs. The control individuals were drawn from 193,150 elderly men without cancer in the 5% sample of Medicare beneficiaries residing in the SEER areas between 1992 and 2007*.* The final cohort of elderly men (≥66 years) diagnosed with prostate cancer was 100,874 patients and the final control cohort of men without prostate cancer was 144,816 patients [6]*.* We excluded men who did not receive treatment for prostate cancer as a possible control group because it is a heterogeneous group (very healthy men that decide to not treat their cancer or very unhealthy men that will die of something else before prostate cancer, so, they decide not to treat it). In addition, it is very difficult to account for “selection by indication” bias and make any generalizations using this group as controls. The number of prostate cancer patients that received each treatment are as follows: 44,318 patients received external beam radiotherapy; 14,259 patients received brachytherapy; 11,835 patients received brachytherapy and external beam radiotherapy; 26,790 patients received radical prostatectomy; 1557 patients received radical prostatectomy and external beam radiotherapy; 2115 patients received cryotherapy.

**Table 1:** Death rates and cumulative incidence functions stratified by treatment group^a^

	Control (*n* = 144,816)	EBRT (*n* = 44,318)	BT (*n* = 14,259)	BT + EBRT (*n* = 11,835)	RP (*n* = 26,790)	RP + EBRT (*n* = 1557)	Cryotherapy (*n* = 2115)
Number of deaths (%)	44,955 (31.0)	10, 522 (23.7)	1830 (12.8)	1658 (14.0)	3365 (12.6)	162 (10.4)	192 (9.1)
KM 10-yr. CIF(%)	50.5	44.0	30.1	30.5	19.3	23.8	32.1
95% Confidence Interval for KM 10-yr. CIF	50.1, 50.9	43.1, 44.8	28.4, 31.8	28.8, 32.2	18.6, 20.1	19.8, 28.0	25.4, 39.0

*Abbreviations*: *KM* Kaplan-Meier, *CR* competing risks, *HR* hazard ratio, *CI* confidence interval

^a^ *EBRT* external beam radiotherapy, *BT* brachytherapy, *RP* radical prostatectomy

**Table 2:** Un-weighted and IPT-weighted Kaplan-Meier and competing risk of any urinary adverse event

	Control	EBRT	BT	BT + EBRT	RP	RP + EBRT	Cryotherapy
Subjects (*n*)	144,816	44,318	14,259	11,835	26,790	1557	2115
Un-weighted KM^a^							
Event rate (n per 100 person-yr)	1.78	2.40	2.70	3.91	4.02	6.08	3.71
KM 10-yr. cumulative incidence (%)	16.1	19.7	19.8	28.4	26.6	37.8	23.4
IPT-weighted KM^a^							
KM 10-yr. cumulative incidence (%)	17.0	17.6	20.0	27.4	27.2	36.4	19.4
HR^b^	1	1.114	1.428	1.969	2.442	3.194	1.56
95% CI		1.07, 1.16	1.33, 1.53	1.85, 2.10	2.34, 2.55	2.79, 3.66	1.30, 1.87
Un-weighted CR							
KM 10-yr. cumulative incidence (%)	12.1	16.4	18.1	25.8	25.6	36.2	22.4
HR^c^	1	1.430	1.660	2.416	2.835	4.057	2.082
95% CI		1.38, 1.48	1.58, 1.74	2.31, 2.53	2.75, 2.93	3.70, 4.46	1.85, 2.35
IPT-weighted CR							
KM 10-yr. cumulative incidence (%)	12.7	14.8	18.3	25.2	26.0	36.5	22.1
HR^d^	1	1.194	1.585	2.214	2.761	3.655	1.754
95% CI		1.15, 1.24	1.50, 1.67	2.11, 2.32	2.67, 2.86	3.28, 4.07	1.54, 1.99

*Abbreviations*: *EBRT* external beam radiotherapy, *BT* brachytherapy, *RP* radical prostatectomy, *UAE* urinary adverse event, *KM* Kaplan-Meier, *CR* competing risks, *HR* hazard ratio, *CI* confidence interval

^a^Results from the Jarosek et al. paper for un-weighted KM and IPT-weighted KM models

^b^Cox proportional hazard model with inverse probability weighting

^c^Competing risks analysis model

^d^Competing risks analysis model with inverse probability of treatment weighting

All treatments were essentially done one time with sometime between therapies for combination treatments. The survival time to UAE for cases is time since prostate cancer treatment to UAE. All controls were assigned pseudo-diagnosis dates as defined in Jaorsek et al. [6]. The survival time to UAE for controls is time since pseudo-diagnosis date to UAE. The prognostic variables in the dataset used for the propensity weighting include the variables of age, race, comorbidity, zip code income and education level, region of SEER registry, year of the treatment, and presence of baseline UAEs [6]. All competing risks models with or without propensity-weighting were adjusted for the covariates of age, comorbidity, and baseline UAE to account for potential residual confounding using the same adjustment covariates as in the article by Jarosek et al. [6].

## Methods

### Survival analysis and competing risks

Clinical researchers use the Kaplan-Meier (KM) survival probability estimator when survival analysis is appropriate for clinical outcomes. The survival function is the probability of an individual surviving beyond time *t* (experiencing the event after time *t*). It is defined as *S(t) = P(T > t)*. Let *t*_*j*_ denote the distinct ordered event times, and *d*_*j*_ be the number of events and *n*_*j*_ be the number at risk at time *t*_*j*_. The KM estimator of the survival function *S*_*KM*_(*x*) is defined as 1 if *t < t*_*1*_ and $\prod_{t_j\le t}\left(1-\frac{d_j}{n_j}\right)$ if *t ≥ t*_*1*_ [15]. The probability of failure is the probability that an individual experiences an event before time *t*, defined as *F(t) = 1− S(t) = P(X ≤ t)*. The KM estimator of the failure function *F*_*KM*_(*t*) is commonly defined as 1 − *S*_*KM*_(*t*) [8].

In the presence of competing events when multiple causes of failure are possible, the naïve KM estimator by censoring competing events does not correctly estimate the cumulative incidence function (CIF) for the events of interest (referred to as the KM CIF estimator) [7, 8, 21]. The naïve KM estimator does not correctly estimate the CIF, because the number of subjects that experience the competing events influences the number of subjects that can actually experience the outcome of interest. For example, say the event of interest for a study sample is the first incident of AIDS diagnosis and the competing event is death. Therefore, the cause-specific hazard function for the first incident of AIDS diagnosis is influenced by the competing event of death in terms of a decrease in the number of subjects that are at risk for an AIDS diagnosis rather than just being censored due to lost to follow-up [24]. The *cumulative incidence function* of cause *k*, *I*_*k*_ (*t*), is defined by the probability P*(T ≤ t, D* = *k)* of failing from cause *k* before time *t*. It can be expressed in terms of the cause-specific hazard as *I*_*k*_ (*t*) *=* ${\int}_0^t\lambda (x) S(x) dx$, where *λ*_*k*_ (*t*) is the cause-specific hazard function and *S* (*t*) is the survival probability of an individual surviving any cause of event beyond time *t*. Appropriately accounting for the competing events, the estimation of unweighted and inverse probability of treatment (IPT)-weighted cumulative incidence functions (referred to as the competing risks CIF estimator) are presented in Additional file 2: Appendix B. A hypothetical example is presented in Additional file 1: Appendix A to illustrate the difference between the naïve KM CIF and the competing risks CIF estimators, and between the unweighted and IPT-weighted competing risks CIF estimators. Additional file 3: Appendix C presents detailed SAS code used to perform these various statistical methods.

### Confounding bias and inverse probability of treatment weighting

In clinical observational studies, a goal of the study is often to compare a treatment group with a control group to understand the impact of the treatment on an event of interest. However, the treatment and control groups may not be comparable without randomization. Therefore, demographic and other characteristics may differ by the treatment groups. When there are differences in the treatment groups by factors that are prognostic for the event of interest, any naïve comparison of the treatment groups may not provide a consistent estimator of the actual (or “causal”) effect of the treatment [13, 25]. To minimize the potential for confounding bias within observational studies, an appropriate study design should be considered to measure and adjust for known and suspected confounding variables [26].

There are several competing approaches to account for measured confounding. One may conduct an IPT-weighted analysis, propensity score adjustment, or outcome-model adjustment. We will concentrate on the first, and defer discussion of relative merits of this approach to the conclusion. For each subject within the study sample, a weight is assigned based on the inverse of the probability of the subject’s actual treatment. For example, in the SEER-Medicare dataset, weights were assigned based on the inverse of the probability of the patient being treated by a specific strategy conditional on the patient’s cancer diagnosis and demographics. In the calculation of the weights, a generalized logistic regression model (or other generalized linear models) is fit to estimate the probabilities of being treated [13, 27].

The method of deriving the weights within the dataset began by modeling the propensity of a diagnosis of prostate cancer using the variables of age, race, comorbidity, zip code income and education level, type of SEER registry, year of the treatment, and presence of baseline UAEs. Second, to account for the variables associated with treatment selection among cases with cancer, we modeled the propensity of receiving treatment with clinical T-stage, grade, and the covariates from the previous model. The IPT weight was defined as the stabilized inverse probability of cancer diagnosis for control subjects, and as the product of the stabilized inverse probability of cancer diagnosis and the stabilized inverse probability of receiving treatment for cases [6]. There was truncation at the 99th percentile of the weights to prevent sparse data (weights can be between 0 and infinity so weights that were extreme outliers were deleted) [6, 27]. Overall, the IPT weight adjusted for the imbalance within the treatment and control groups among the baseline and demographic characteristics so that subjects who were least like the others in their treatment group were given higher weighting.

### Statistical analysis

Time to the first event of UAE (event of interest), censoring events (radiation, chemotherapy, and/or surgery), or death (competing event) was defined in days measured from the first claim date of treatment. Stabilized IPT weights to account for confounding bias were calculated by Jarosek et al. given differences in patient and tumor characteristics within each treatment group [6]. IPT-weighted competing risks analysis estimated the hazard ratio of UAEs in general for each treatment group. The cause-specific hazard ratio for UAE was estimated with a 95% confidence interval and the estimated 10-year cumulative incidence of UAE for each competing risks model was also calculated. All statistical analyses were performed using SAS v9.4 TS1M2 (SAS Institute, Inc., Cary, NC, USA).

To help the reader understand the changes in the hazard ratios with the addition of IPT weighting and competing risks analysis, we first compared (no statistical test was used to detect differences) the results of the IPT-weighted competing risks analysis to the un-weighted competing risks analysis. Then we compared the results from the un-weighted Cox models to the IPT-weighted Cox models. The hazard ratio of UAE from the IPT-weighted competing risks analysis was compared to the hazard ratio estimated from the competing risks analysis without IPT weighting. In addition, the hazard ratios of UAEs by un-weighted and IPT-weighted competing risks analysis were compared to the results of the un-weighted and IPT-weighted Cox proportional hazard models estimated by Jarosek et al. (Table 2 of Jarosek et al.).

Death rates among the total cohort were not uniform across all treatment groups. The difference in death rates by prostate cancer treatment provides justification for competing risks analysis because the occurrence of the event types (UAE, death, and censoring events) are mutually exclusive [28]. A standard KM curve was estimated for the death rate by treatment group where any UAE events were censored and the event of interest was death.

## Results

**Fig. 1:**
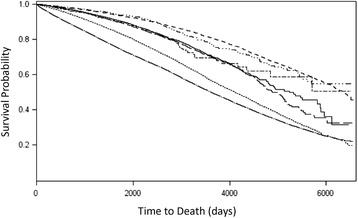
Product-limit survival estimates. Survival probability of death by treatment group. EBRT = external beam radiotherapy; BT = brachytherapy; RP = radical prostatectomy. Treatment groups: : BT; : BT + EBRT; : Control; : Cryotherapy; : EBRT; : RP; : RP + EBRT

Figure 1 displays the survival probability versus time by days to death for each prostate cancer treatment group. The figure shows the survival probability of death by days to event for each prostate cancer treatment group using a Propensity-weighted Cox proportional hazards model with death as the event of interest and all other events as censored observations. The difference in death rates by prostate cancer treatments provides justification for the IPT-weighted competing risks analysis because death precludes observing any urinary adverse events (i.e. mutually exclusive events). The number of death events for each treatment group is shown in Table 1. The treatment group that experienced the highest number of deaths was external beam radiotherapy with a 23.7% death rate. The death rate among the control group (men aged greater than or equal to 66 years old without cancer) was 31.0%. Thus, the 10-yr. cumulative incidence of death was highest in the control group followed by the external beam radiotherapy group and the cryotherapy group.

The results of the two Cox proportional hazard models adjusted using the inverse probability weighting and without inverse probability weighting from Jarosek et al. are provided as reference in Table 2. The interpretation of those models and discussion of the results are found in the article as well.

The 10-yr. competing risks cumulative incidence of a UAE without IPT weighting was highest among the radical prostatectomy + external beam radiotherapy treatment group followed by the brachytherapy + external beam radiotherapy group and the radical prostatectomy group (Table 2). All 10-yr. competing risks cumulative incidences of UAE are lower than the 10-yr. KM cumulative incidences of UAE. After taking into account IPT-weighted competing risks analysis, the 10-yr. cumulative incidence of UAE increased slightly for the radical prostatectomy + external beam radiotherapy group to 36.5% compared to the competing risks cumulative incidence of a UAE without IPT weighting. However, for both IPT-weighted competing risks analysis and non-weighted IPT competing risks analysis, the radical prostatectomy + external beam radiotherapy treatment group remained as having the highest cumulative UAE incidence.

The cumulative risk estimated without IPT weighting at year 10 for UAEs for the external beam radiotherapy and brachytherapy treatment groups alone were much smaller (16.4 and 18.1%, respectively) compared to the brachytherapy + external beam radiotherapy and radical prostatectomy treatment groups (25.8 and 25.6%, respectively). In a similar comparison with the IPT-weighted competing risks analysis, the 10-yr. competing risks cumulative incidence of UAE for the external beam radiotherapy and brachytherapy treatment groups were 14.8 and 18.3%, respectively, which are in the same way much smaller than the brachytherapy + external beam radiotherapy and radical prostatectomy treatment groups (25.2 and 26.0%, respectively).

The competing risks model without IPT weighting for the estimated time for a patient’s first UAE showed an increased risk for all treatment groups compared to the control group (Table 2). The treatment group, radical prostatectomy + external beam radiotherapy, experienced the largest increased risk for UAE compared to the control group (hazard ratio [HR] 4.06 with 95% confidence interval [CI]: 3.70, 4.46). The treatment group with the lowest increased risk for a UAE compared to the control group was the external beam radiotherapy group (HR 1.43 with 95% CI: 1.38, 1.48).

The IPT-weighted competing risks model predicting time to first UAE indicated increased risk of a UAE for all treatment groups compared to the control group (Table 2). Patients within the radical prostatectomy + external beam radiotherapy treatment group experienced the highest increased risk for a UAE (HR 3.66 with 95% CI: 3.28, 4.07). The treatment group that had the lowest increased risk for UAE compared to the control group was the external beam radiotherapy group (HR 1.19 with 95% CI: 1.15, 1.24). Overall, all the treatment groups experienced a higher increased risk for UAE with all hazard ratios from the IPT-weighted competing risks model being larger compared to the hazard ratio estimates from the IPT-weighted Cox proportional hazards model.

## Discussion

Competing risks influences the cumulative incidence of a UAE by removing the expected number of subjects that die (the competing event) from the calculation of the survival probability (KM estimate of UAE and death-free survival). Subjects that experience the competing event are not counted in the potential group of subjects that could experience a UAE. Competing risks are prevalent in many types of clinical research where the probability of an event of interest is potentially biased due to the presence of another event attributed either to the nature of the disease or the treatment option.

Competing risks analysis is underused in clinical research, but in particular rarely used in combination with IPT-weighted models for survival analysis. When there is a potential for confounding bias, IPT weighting can take into account the probability of treatment selection based on various factors of interest. IPT-weighted competing risks analysis provides a consistent estimate of the ratio of marginal hazards through the hazard ratio. In addition, an IPT-weighted competing risk analysis provides an unbiased estimate of the cumulative incidence of the event of interest in the presence of competing risks, while neither a KM curve nor an IPT-weighted KM do so. In some studies, it is possible that the confounding factors are not identical for all causes (i.e., some confounding factors might only relate to the CIF for a particular cause). Ideally, one might incorporate this difference in the estimation of weights and IPT-weighted competing risks analysis. However, this can be statistically challenging and corresponding statistical theory and software remain to be developed. As a practical solution, if the union of the confounding factors from all causes are included in the weight estimation (and no collider-stratification bias is induced), we obtain a consistent estimate of the risks, but inclusion of unnecessary variables may cost some statistical efficiency.

Using the matched-cohort prostate cancer dataset from Jarosek et al., the addition of the un-weighted and IPT-weighted competing risks analysis was provided for comparison to the Cox proportional hazard models to demonstrate the difference in 10-yr. cumulative incidence estimates for UAE as well as the risk of UAE for each treatment group compared to the control group. The death rate was much higher among the control group than the cancer treatment group. This is counter-intuitive but may be due to a bias to screen healthy men for prostate cancer and not to screen unhealthy men.

To investigate the comparison between the hazard ratios for the IPT-weighted Cox proportional hazards model and the IPT-weighted competing risks model, a Cox proportional hazards model was constructed with death as the event of interest and all other events including a UAE event were censored. A hazard ratio was calculated for each treatment group compared to the control group (results not shown). For all treatment groups, there was a decreased risk for death compared to the control group. The reasoning for this may be a combination of: 1. how the control group of non-cancer elderly men was assigned pseudo diagnosis dates to correctly assign the appropriate number of control subjects for each diagnosis month and 2. the fact that prostate cancer patients who were treated may be a bit healthier than some prostate cancer patients who were not treated. Because the control group was the reference population, the analyses that did not account for competing risks of death significantly underestimated the hazard ratio of UAEs. Overall, men with prostate cancer need to understand that any survival benefit of treatment will also come with the tradeoff of possible long term sequelae. Our current analysis provides a higher, and perhaps more accurate, estimate of the hazard ratio of UAEs among men treated for prostate cancer.

There are limitations of this secondary data analysis. These include the inability to calculate standard error estimates and consequently 95% confidence intervals for the 10-yr. cumulative incidence of UAE for all model estimates. The use of PROC PHREG can compute standard errors through the option cif = _all_. However, the memory required to compute a standard error for each cumulative incidence estimate without re-calculating the standardized inverse probability weight for each observation was too much for the computing power given the resources allocated for this analysis (Windows Base SAS, 8GB memory, and Intel Pentium processor). Future research should look into alternative methods to calculate the standard error using nonparametric methods such as bootstrapping. Jarosek et al. provides the limitations of the generalizability of the results to prostate cancer patients receiving treatment.

## Conclusions

In clinical cancer research, IPT-weighted competing risks analysis should be used to account for differences in treatments and the competing risk of an event such as death. This type of analysis allows for accurate and unbiased estimates of the cumulative incidence and risk of the event of interest. This paper illustrated how to understand the 10-year risks of UAEs among men diagnosed with prostate cancer that were treated with various therapies taking into account the competing risk of death. Furthermore, this paper presents a tutorial for clinicians and non-statistical researchers to facilitate their own application of IPT-weighted competing risks analysis.

## Additional files

Additional file 1: Appendix A: The Estimation of Cumulative Incidence Function. This appendix provides the equations for estimating the un-weighted and weighted cumulative incidence functions. (PDF 356 kb)

Additional file 2: Appendix B: A Hypothetical Example. This example of 10 patients illustrates the detailed calculations of the various methods (Competing Risks, Kaplan Meir, un-weighted and weighted methods). Also, the differences between the multiple methods for estimating the cumulative incidence function are highlighted. (PDF 240 kb)

Additional file 3: Appendix C: SAS Code. This appendix provides the SAS code to perform the various analysis methods in Appendix B as well as the paper: un-weighted Kaplan Meir, weighted Kaplan Meir, un-weighted Competing Risks, weighted Competing Risks, and IPT-weighted Competing Risks. (PDF 93 kb)

## Abbreviations

CI: Confidence interval
CIF: Cumulative incidence function
HR: Hazard ratio
IP: Inverse probability
IPT: Inverse probability of treatment
KM: Kaplan-Meier
SEER: Surveillance, Epidemiology and End Results
UAEs: Urinary adverse events
