# Supplementary material for: Inverse probability of treatment-weighted competing risks analysis: an application on long-term risk of urinary adverse events after prostate cancer treatments
Source: BMC Med Res Methodol. 2017 Jul 10;17:93. doi: 10.1186/s12874-017-0367-8 (PMC5504854; doi:10.1186/s12874-017-0367-8)
Supplement: Supplementary file 1 — Appendix A: The Estimation of Cumulative Incidence Function. This appendix provides the equations for estimating the un-weighted and weighted cumulative incidence functions. (PDF 356 kb) [file 12874_2017_367_MOESM1_ESM.pdf]

Appendix A: The Estimation of Cumulative Incidence Function

Let us consider a competing risk time-to-event study with *N* independent observations concerning the situation where multiple causes of failure (*K*) are possible. Let w_i be the inverse probability (IP) weight for the ith subject ($i = 1, 2, \ldots, N$). Let $0 < t_1 < t_2 < \ldots < t_j$ denote the ordered distinct event time points at which failures of any cause occur. Let d_{kj} denote the number of patients failing from cause *k* ($k = 1, 2, \ldots, K$) at t_J and R_{kj} be the corresponding set of individuals. Let $d_j = \sum_{k=1}^{K} d_{kj}$ denote the total number of failures from any cause. Let n_j be the number of individuals at risk at time t_j and R_j be the corresponding set of individuals. Thus, the weighted number of events failing from cause k is $d_{kj}^{W} = \sum_{i \in R_{kj}} w_i$, the weighted total number of failures from any cause is $d_j^{W} = \sum_{k=1}^{K} d_{kj}^{W}$, and the weighted number of individuals at risk is $n_j^{W} = \sum_{i \in R_j} w_i$ at time t_j.

The overall unweighted survival probability $S(x)$, can be estimated by the Kaplan-Meier estimator as $\hat{S}(x) = \prod_{j:\, t_j \leq x} \left(1 - \frac{d_j}{n_j}\right) = \prod_{j:\, t_j \leq x} \left(1 - \sum_{k=1}^{K} \widehat{\lambda_k}\ (t_j)\right)$, where the unweighted cause specific hazard function $\widehat{\lambda_k}\ (t_j) = {d_{kj}}/{n_j}$. The unweighted cumulative incidence $I_k(x)$ of cause *k* at time *x* is estimated $\widehat{I_k}(x) = \sum_{j:\, t_j \leq x} \widehat{\lambda_k}\ (t_j)\ \hat{S}\ (t_{j-1})$.

The overall inverse probability of treatment (IPT)-weighted survival probability $S^{W}(x)$, can be estimated by the weighted Kaplan-Meier estimator as $\widehat{S^{W}}(x) = \prod_{j:\, t_j \leq x} \left(1 - \frac{d_j^{W}}{n_j^{W}}\right) = \prod_{j:\, t_j \leq x} \left(1 - \sum_{k=1}^{K} \widehat{\lambda_j^{W}}\ (t_j)\right)$, where the IPT-weighted cause specific hazard function $\widehat{\lambda_k^{W}}\ (t_j) = {d_{kj}^{W}}/{n_j^{W}}$. The weighted cumulative incidence $I_k^{W}\ (x)$ of cause k at time *x* is estimated $\widehat{I_k^{W}}(x) = \sum_{j:\, t_j \leq x} \widehat{\lambda_k^{W}}\ (t_j)\ \widehat{S^{W}}\ (t_{j-1})$.
