# Supplementary material for: Inverse probability of treatment-weighted competing risks analysis: an application on long-term risk of urinary adverse events after prostate cancer treatments
Source: BMC Med Res Methodol. 2017 Jul 10;17:93. doi: 10.1186/s12874-017-0367-8 (PMC5504854; doi:10.1186/s12874-017-0367-8)
Supplement: Supplementary file 2 — Appendix B: A Hypothetical Example. This example of 10 patients illustrates the detailed calculations of the various methods (Competing Risks, Kaplan Meir, un-weighted and weighted methods). Also, the differences between the multiple methods for estimating the cumulative incidence function are highlighted. (PDF 240 kb) [file 12874_2017_367_MOESM2_ESM.pdf]

**Appendix B: A Hypothetical Example**

In this example, we show the calculations for the cumulative incidence using the competing risks and Kaplan-Meier (KM) methods, and the un-weighted and weighted competing risks methods. The formulas for all calculations are in Appendix A. A sample of 10 patients was generated in SAS with the following event or censored times as shown below:

10(U), 15(D), 20(U), 30(U), 35+, 45(D), 50(U), 55(D), 75(U), 90+

A censored time is denoted by (+), (U) denotes a urinary adverse event, and (D) denotes death. The computation for the KM estimate of cumulative incidence for a urinary adverse event (UAE) and the competing risks estimate of the cumulative incidence of a UAE is shown in Table 3. At the start of the study, all patients were alive so the KM estimate for UAE-free survival at t = 0 is 1 and the KM estimate for the cumulative incidence of UAE is 0. If one UAE happens at $t_1$ = 10 (after 10 months of follow-up time), then the KM estimate for UAE-free survival is defined as $\widehat{S}$ (*x* = 10) = $\prod_{1:t_1 \leq 10}\left(1-\frac{1}{10}\right)$ = 0.9. The KM cumulative incidence function (CIF) for UAE at $t_1$ = 10 is defined as $\widehat{I_2}$ (*x* = 10) $= \frac{1}{10} \times 1 = 0.1$. The next event of death occurs at $t_6$ = 45 so the KM UAE-free survival decreases to 0.7 and the KM CIF of UAE is 0.32. Since the last patient did survive past $t_{10}$ = 90, the KM estimate for UAE-free survival is 0.25 and the KM CIF of UAE remained at 0.75.

In contrast to the KM estimate of the cumulative incidence of UAE, Table 3 also displays the computations for the competing risks estimate of cumulative incidence of UAE. The first UAE is at $t_1$ = 10, so the KM estimate of UAE/death-free survival is 0.9 and the competing risks CIF of UAE is 0.1. At $t_3$ = 20, the patient experiences a UAE so the KM estimate of UAE/death-free survival is calculated as $\widehat{S}$ (*x* = 20) = $(1 - \frac{1}{10}) \times (1-\frac{1}{9}) \times (1-\frac{1}{8})$ = 0.7; but the competing risks

CIF of UAE increases because $\widehat{I_2}$ ($x$ = 20) = $\left(\frac{1}{10}\times 1\right)+\left(\frac{0}{9}\times 0.9\right)+\left(\frac{1}{8}\times 0.8\right)$ = 0.2. The final competing risks CIF of UAE at $t_{10}$ = 90 was 0.54 which is smaller than the KM CIF of UAE due to the fact that the KM method overestimated the cumulative incidence of UAE.

The calculations in Table 4 for the cumulative incidence of UAEs for the weighted and un-weighted competing risks methods are contrasted to the computations in Table 3. The estimates are labeled as un-weighted in Table 4 to differentiate the weighted and un-weight methods for calculating the competing risks CIF of UAE. The weights for Table 4 were randomly selected between the values of 0.5 and 1.5. Similarly to Table 3, at the start of the study, all patients were alive so the weighted KM estimate for UAE/death-free survival at $t_0$ = 0 is 1 and the weighted competing risks CIF of UAE is 0. At $t_1$ = 10, there was a UAE event so the weighted KM estimate for UAE/death-free survival was decreased to 0.9 and the weighted competing risks CIF of UAE increased to 0.1 which are equal to the calculations for the un-weighted estimates. Another UAE event with a weight of 1.4 was observed at 30 months which means the weighted KM estimate of UAE/death-free survival is defined as $\widehat{S^w}$ ($x$ = 30) = $(1-\frac{0.9}{9.7})\times(1-\frac{1}{8.8})\times(1-\frac{0.7}{7.8})\times(1-\frac{1.4}{7.1})$ = 0.6. The weighted competing risks CIF of UAE increased to 0.33 because $\widehat{I_2^w}$($x$ = 30) = $\left(\frac{0.9}{9.7}\times 1\right)+\left(\frac{0}{8.8}\times 0.9\right)+\left(\frac{0.7}{7.8}\times 0.8\right)+\left(\frac{1.4}{7.1}\times 0.7\right)\cong 0.33$. At the final time of 90 months, the patient was still alive so the weighted KM estimate for UAE/death-free survival remained at 0.2. The weighted competing risks CIF of UAE was 0.59, which was 0.05 units higher than the un-weighted competing risks CIF of UAE.

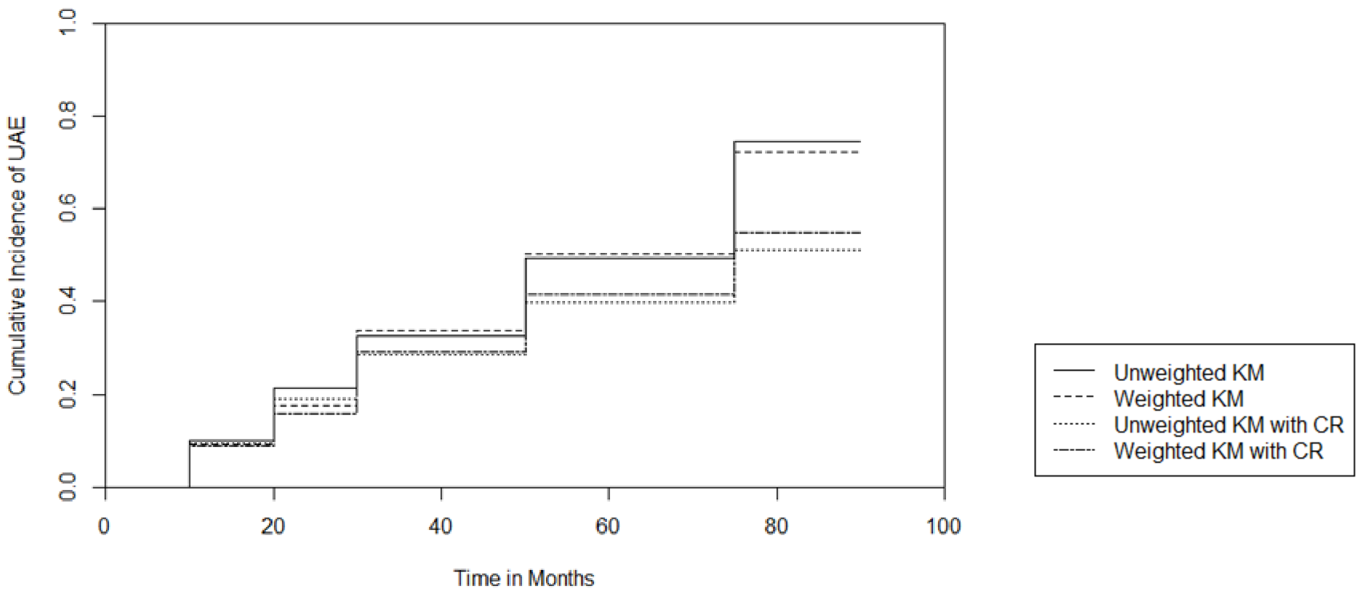


**Figure 2:** Kaplan-Meier vs. Competing Risks CIF Estimates with Weighting and No Weighting

Four cumulative incidence function (CIF) curves calculated in Table 3 and 4: Un-weighted Kaplan-Meier (KM) method for CIF, Weighted KM method for CIF, Un-weighted competing risks method for CIF, and IPT-weighted competing risks method for CIF.

Figure 2 shows the CIF for the competing risks and KM methods which illustrate the differences in estimates of the cumulative incidence of UAE from the hypothetical example. The four different curves represent the cumulative incidence of UAE calculated in Table 3 and 4: un-weighted KM method, un-weighted competing risks method, weighted competing risks method, and the weighted KM method (not shown in Table 3 or 4). The highest CIF of UAE at 90 months was 0.75 and estimated by the un-weighted KM method. The weighted KM method estimated the CIF of UAE at 90 months to be 0.72, which was slightly lower taking into account weighting. The un-weighted competing risks method estimated the CIF of UAE to be 0.54 and the weighted CR method at 0.59. The difference between the weighted KM and weighted competing risks is 0.13, which concludes that taking into account IPT weighting, and competing risks provides a more accurate estimate of the cumulative incidence of the event.

**Table 3.** Cumulative Incidence of Urinary Adverse Events: Competing Risks and Kaplan-Meier Methods

| Patient No. | Follow-up time ($t_j$) | Event ($k$) | No. at risk ($n_j$) | No. of urinary adverse effects ($d_j$) | No. of censored observations for KM method | KM estimate for UAE-free survival $\hat{S}(x)$ | KM estimate of cumulative incidence of UAE $\hat{I}_{k=2}(x)$ | No. of censored observations for CR method | KM estimate for UAE and death-free survival $\hat{S}(x)$ | CR estimate of cumulative incidence of UAE $\hat{I}_{k=2}(x)$ |
|---|---|---|---|---|---|---|---|---|---|---|
| 1 | 10 | UAE | 10 | 1 | 0 | 1 x (9/10) = 0.9 | 0 + 1 x (1/10) = 0.1 | 0 | 1 x (9/10) = 0.9 | 0 + 1 x (1/10) = 0.1 |
| 2 | 15 | Death | 9 | 0 | 1 | 0.9 x (9/9) = 0.9 | 0.1 + 0.9 x (0/9) = 0.1 | 0 | 0.9 x (8/9) = 0.8 | 0.1 + 0.9 x (0/9) = 0.1 |
| 3 | 20 | UAE | 8 | 1 | 0 | 0.9 x (7/8) = 0.8 | 0.1 + 0.9 x (1/8) = 0.21 | 0 | 0.8 x (7/8) = 0.7 | 0.1 + 0.8 x (1/8) = 0.2 |
| 4 | 30 | UAE | 7 | 1 | 0 | 0.8 x (6/7) = 0.7 | 0.21 + 0.8 x (1/7) = 0.32 | 0 | 0.7 x(6/7) = 0.6 | 0.2 + 0.7 x (1/7) = 0.3 |
| 5 | 35+ | Alive | 6 | 0 | 1 | 0.7 x (6/6) = 0.7 | 0.32 + 0.7 x (0/6) = 0.32 | 1 | 0.6 x (6/6) = 0.6 | 0.3 + 0.6 x (0/6) = 0.3 |
| 6 | 45 | Death | 5 | 0 | 1 | 0.7 x (5/5) = 0.7 | 0.32 + 0.7 x (0/5) = 0.32 | 0 | 0.6 x (4/5) = 0.48 | 0.3 + 0.6 x (0/5) = 0.3 |
| 7 | 50 | UAE | 4 | 1 | 0 | 0.7 x (3/4) = 0.5 | 0.32 + 0.7 x (1/4) = 0.5 | 0 | 0.48 x (3/4) = 0.36 | 0.3 + 0.48 x (1/4) = 0.42 |
| 8 | 55 | Death | 3 | 0 | 1 | 0.5 x (3/3) = 0.5 | 0.5 + 0.5 x (0/3) = 0.5 | 0 | 0.36 x (2/3) = 0.24 | 0.42 + 0.36 x (0/3) = 0.42 |
| 9 | 75 | UAE | 2 | 1 | 0 | 0.5 x (1/2) = 0.25 | 0.5 + 0.5 x (1/2) = 0.75 | 0 | 0.24 x (1/2) = 0.12 | 0.42 + 0.24 x (1/2) = 0.54 |
| 10 | 90+ | Alive | 1 | 0 | 1 | 0.25 x (1/1) = 0.25 | 0.75 + 0.25 x (0/1) = 0.75 | 1 | 0.12 x (1/1) = 0.12 | 0.54 + 0.12 x (0/1) = 0.54 |

**Table 4.** Cumulative Incidence of Urinary Adverse Events: Un-Weighted and Weighted Competing Risks

| Patient No. | Follow-up time ($t_j$) | Event ($k$) | No. at risk ($n_j$) | No. of urinary adverse effects ($d_j$) | No. of censored observations | Weight ($w_i$) | Un-weighted KM estimate for UAE and death-free survival $\hat{S}(x)$ | Un-weighted CR estimate of cumulative incidence of UAE $\hat{I}_{k=2}(x)$ | Weighted KM estimate for UAE and death-free survival $\widehat{S^w}(x)$ | Weighted CR estimate of cumulative incidence of UAE $\hat{I}_{k=2}{}^{w}(x)$ |
|---|---|---|---|---|---|---|---|---|---|---|
| 1 | 10 | UAE | 10 | 1 | 0 | 0.9 | 1 x (9/10) = 0.9 | 0 + 1 x (1/10) = 0.1 | 1.0 x (8.8/9.7) = 0.9 | 0 + 1 x (0.9/9.7)= 0.1 |
| 2 | 15 | Death | 9 | 0 | 0 | 1 | 0.9 x (8/9) = 0.8 | 0.1 + 0.9 x (0/9) = 0.1 | 0.9 x (7.8/8.8) = 0.8 | 0.1 + 0.9 x (0/8.8) = 0.1 |
| 3 | 20 | UAE | 8 | 1 | 0 | 0.7 | 0.8 x (7/8) = 0.7 | 0.1 + 0.8 x (1/8) = 0.2 | 0.8 x (7.1/7.8) = 0.7 | 0.1 + 0.8 x (0.7/7.8) = 0.17 |
| 4 | 30 | UAE | 7 | 1 | 0 | 1.4 | 0.7 x (6/7) = 0.6 | 0.2 + 0.7 x (1/7) = 0.3 | 0.7 x (5.7/7.1) = 0.6 | 0.2 + 0.7 x (1.4/7.1) = 0.33 |
| 5 | 35+ | Alive | 6 | 0 | 1 | 0.8 | 0.6 x (6/6) = 0.6 | 0.3 + 0.6 x (0/6) = 0.3 | 0.6 x (5.7/5.7) = 0.6 | 0.33 + 0.6 x (0/5.7) = 0.33 |
| 6 | 45 | Death | 5 | 0 | 0 | 0.5 | 0.6 x (4/5) = 0.48 | 0.3 + 0.6 x (0/5) = 0.3 | 0.6 x (4.4/4.9) = 0.5 | 0.33 + 0.6 x (0/4.9) = 0.33 |
| 7 | 50 | UAE | 4 | 1 | 0 | 1.1 | 0.48 x (3/4) = 0.36 | 0.3 + 0.48 x (1/4) = 0.42 | 0.5 x (3.3/4.4) = 0.4 | 0.33 + 0.5 x (1.1/4.4) =0.46 |
| 8 | 55 | Death | 3 | 0 | 0 | 0.6 | 0.36 x (2/3) = 0.24 | 0.42 + 0.36 x (0/3) = 0.42 | 0.4 x (2.7/3.3) = 0.3 | 0.46 + 0.4 x (0/3.3) = 0.46 |
| 9 | 75 | UAE | 2 | 1 | 0 | 1.2 | 0.24 x (1/2) = 0.12 | 0.42 + 0.24 x (1/2) = 0.54 | 0.3 x (1.5/2.7) = 0.2 | 0.46 + 0.3 x (1.2/2.7) = 0.59 |
| 10 | 90+ | Alive | 1 | 0 | 1 | 1.5 | 0.12 x (1/1) = 0.12 | 0.54 + 0.12 x (0/1) = 0.54 | 0.2 x (1.5/1.5) = 0.2 | 0.59 + 0.2 x (0/1.5) = 0.59 |
