# Supplementary material for: Inverse probability of treatment-weighted competing risks analysis: an application on long-term risk of urinary adverse events after prostate cancer treatments
Source: BMC Med Res Methodol. 2017 Jul 10;17:93. doi: 10.1186/s12874-017-0367-8 (PMC5504854; doi:10.1186/s12874-017-0367-8)
Supplement: Supplementary file 3 — Appendix C: SAS Code. This appendix provides the SAS code to perform the various analysis methods in Appendix B as well as the paper: un-weighted Kaplan Meir, weighted Kaplan Meir, un-weighted Competing Risks, weighted Competing Risks, and IPT-weighted Competing Risks. (PDF 93 kb) [file 12874_2017_367_MOESM3_ESM.pdf]

**Appendix C: SAS Code**

**C.1 SAS Code for the Hypothetical Example Calculations of Competing Risks and KM Cumulative Incidence Estimations of UAE:**

```
*Table 3: Unweighted KM versus unweighted CIF;
*Table 4: Weighted KM versus weighted CIF;

*Dataset of 10 patients;
*The variable Ftime represents the disease-free survival in months, which is the time to UAE,
time to death, or censored;
*The variable Status is an indicator with the value 0 for censoring, 1 for UAE, or 2 for death
during remission;

*Dataset for Table 3 and 4;
data prostate_cancer;
	input PatientID $ Ftime Event $ At_Risk CR_UAE KM_UAE Weight;
	datalines;
1 10 UAE 10 1 1 0.9
2 15 Death 9 2 0 1
3 20 UAE 8 1 1 0.7
4 30 UAE 7 1 1 1.4
5 35 Alive 6 0 0 0.8
6 45 Death 5 2 0 0.5
7 50 UAE 4 1 1 1.1
8 55 Death 3 2 0 0.6
9 75 UAE 2 1 1 1.2
10 90 Alive 1 0 0 1.5
;

*Un-weighted Competing Risks Method;
*With event=1 for UAE;
PROC PHREG DATA=prostate_cancer plots=cif COVS(AGGREGATE);
MODEL Ftime*CR_UAE(0)= / eventcode=1;
BASELINE OUT=out_CRNW CIF=_ALL_;
RUN;

*Un-weighted KM Method;
PROC LIFETEST DATA=prostate_cancer outsurv=out_KMNW plots=survival(failure);
Time Ftime*KM_UAE(0);
title 'KM Method for Unweighted Data';
RUN;
DATA out_KMNW;
SET out_KMNW;
failure = 1-SURVIVAL;
RUN;
```

```
*Weighted KM Method;
PROC LIFETEST DATA=prostate_cancer outsurv=out_KMW;
TIME Ftime*KM_UAE(0);
Weight Weight;
title "KM Method for Weighted Data";
RUN;
DATA out_KMW;
SET out_KMW;
failure = 1-SURVIVAL;
RUN;

*Weighted Competing Risks Method;
PROC PHREG DATA=prostate_cancer plots=cif COVS(AGGREGATE);
MODEL Ftime*CR_UAE(0) = / eventcode=1;
BASELINE OUT=out_CRW CIF=_ALL_;
Weight Weight;
RUN;
```

**C.2 SAS Code for IPT-Weighted and Un-Weighted Competing Risks Analysis:**

```
*Un-weighted competing risks method only adjusted for treatment group;
proc phreg data=prostate_uae_edit COVS(AGGREGATE);
        class txgroup (order=internal ref='control');
        model daystoevent*censor2(2) = txgroup / eventcode=0;
        Hazardratio 'Pairwise' txgroup / diff=pairwise;
        baseline out=out_unweightedCRadju;
run;

*IPT-weighted CR Method only adjusted for treatment group;
proc phreg data=prostate_uae_edit COVS(AGGREGATE);
        class txgroup (order=internal ref='control');
        model daystoevent*censor2(2) = txgroup / eventcode=0;
        Hazardratio 'Pairwise' txgroup / diff=pairwise;
        baseline out=out_weightCRadju;
        weight ipw_st;
run;

*Subset data by treatment group and run unadjusted competing risks model to calculate CIF;
*Example of one treatment group = brybeam;
*brybeam CIF unweighted;
proc phreg data=brybeam COVS(AGGREGATE);
        model daystoevent*censor2(2) = / eventcode=0;
        baseline out=out_unweightCR_brybeam cif=cif;
run;
```

```
*brybeam CIF IPT-weighted;
proc phreg data=brybeam COVS(AGGREGATE);
	model daystoevent*censor2(2) = / eventcode=0;
	baseline out=out_weightCR_brybeam cif=cif;
	weight ipw_st;
run;
```
